# Supplementary material for: Reliability, validity and discriminability of patient reported outcomes for non-specific low back pain in a nationwide physical therapy registry: A retrospective observational cohort study
Source: PLoS One. 2021 Jun 3;16(6):e0251892. doi: 10.1371/journal.pone.0251892 (PMC8174721; doi:10.1371/journal.pone.0251892)
Supplement: S1 Appendix — (DOCX) [file pone.0251892.s001.docx]

# S1 Appendix.

The calculation used to define a more specific sample size, using the equation of Twisk et al. (2013).^40^

| Equation to calculate the sample size by Twisk et al. (2013).^40^  $m=\frac{N}{(1+(n-1)(1-\rho)}$  $N_{effective}=m*n$  m= number of practices  *N*= number of patients according to the rule of thumb.^24,38^  $n$= number of patients for each practice according to the rule of thumb.^24,38^  Ρ= Interclass Correlation Coefficient (ICC)  *N_effective_*= Effective sample size by a given “general” sample size  Sample-size for the domain pain intensity:  $m=\frac{900}{(1+(30-1)(1-0.55)}=64.05\approx65 practices$  $N_{effective}=64.05*30=1921.5\approx1922 patients$  Sample-size for the domain physical functioning:  $m=\frac{900}{(1+(30-1)(1-0.56)}=65.41\approx66 practices$  $N_{effective}=65.41*30=1962.2\approx1963 patients$ |
| --- |
