# Supplementary figures and images for: Reliability, validity and discriminability of patient reported outcomes for non-specific low back pain in a nationwide physical therapy registry: A retrospective observational cohort study
Source: PLoS One. 2021 Jun 3;16(6):e0251892. doi: 10.1371/journal.pone.0251892 (PMC8174721; doi:10.1371/journal.pone.0251892)

S1 Fig. A flowchart of the patient inclusion.


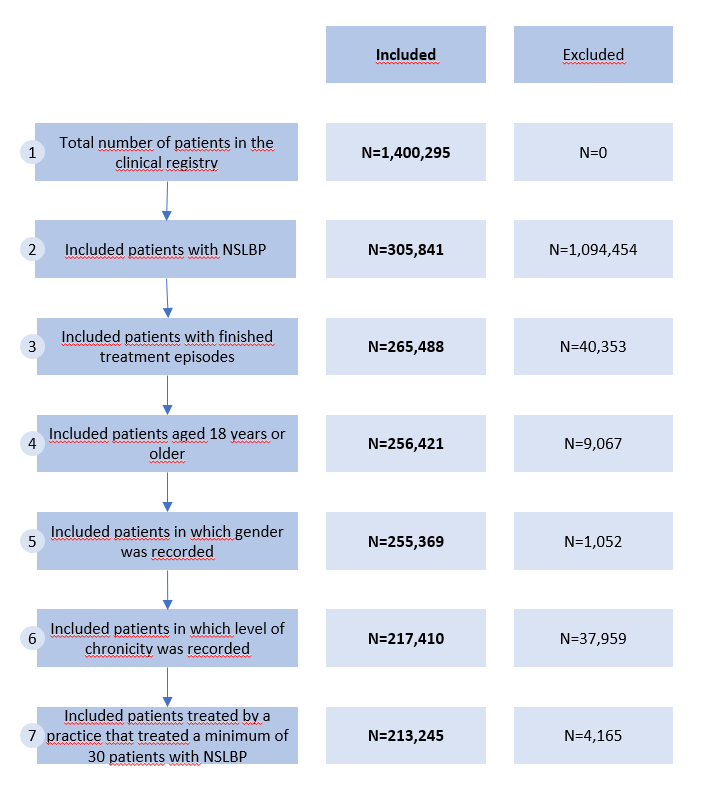

Supplement: S1 Fig — (DOCX) [file pone.0251892.s002.docx]
